# Transpresentation of interleukin-15 by IL-15/IL-15Rα mRNA-engineered human dendritic cells boosts antitumoral natural killer cell activity

**DOI:** 10.18632/oncotarget.6536

**Published:** 2015-12-09

**Authors:** Johan Van den Bergh, Yannick Willemen, Eva Lion, Heleen Van Acker, Hans De Reu, Sébastien Anguille, Herman Goossens, Zwi Berneman, Viggo Van Tendeloo, Evelien Smits

**Affiliations:** ^1^ Laboratory of Experimental Hematology, Vaccine and Infectious Disease Institute (VAXINFECTIO), Faculty of Medicine and Health Sciences, University of Antwerp, Antwerp, Belgium; ^2^ Laboratory of Medical Microbiology, Vaccine and Infectious Disease Institute (VAXINFECTIO), Faculty of Medicine and Health Sciences, University of Antwerp, Antwerp, Belgium; ^3^ Center for Oncological Research Antwerp, Faculty of Medicine and Health Sciences, University of Antwerp, Antwerp, Belgium

**Keywords:** interleukin-15 transpresentation, IL-15 receptor α, dendritic cells, mRNA elektroporation, natural killer cells, Immunology and Microbiology Section, Immune response, Immunity

## Abstract

In cancer immunotherapy, the use of dendritic cell (DC)-based vaccination strategies can improve overall survival, but until now durable clinical responses remain scarce. To date, DC vaccines are designed primarily to induce effective T-cell responses, ignoring the antitumor activity potential of natural killer (NK) cells. Aiming to further improve current DC vaccination outcome, we engineered monocyte-derived DC to produce interleukin (IL)-15 and/or IL-15 receptor alpha (IL-15Rα) using mRNA electroporation. The addition of IL-15Rα to the protocol, enabling IL-15 transpresentation to neighboring NK cells, resulted in significantly better NK-cell activation compared to IL-15 alone. Next to upregulation of NK-cell membrane activation markers, IL-15 transpresentation resulted in increased NK-cell secretion of IFN-γ, granzyme B and perforin. Moreover, IL-15-transpresenting DC/NK cell cocultures from both healthy donors and acute myeloid leukemia (AML) patients in remission showed markedly enhanced cytotoxic activity against NK cell sensitive and resistant tumor cells. Blocking IL-15 transpresentation abrogated NK cell-mediated cytotoxicity against tumor cells, pointing to a pivotal role of IL-15 transpresentation by IL-15Rα to exert its NK cell-activating effects. In conclusion, we report an attractive approach to improve antitumoral NK-cell activity in DC-based vaccine strategies through the use of IL-15/IL-15Rα mRNA-engineered designer DC.

## INTRODUCTION

As the main orchestrators of the immune system, dendritic cells (DC) are ideal candidates in the design of immunotherapeutic strategies to treat cancer patients. For this reason, DC based vaccination is being explored to improve clinical outcome of various cancer patients. As reported by our and other research groups, DC based immunotherapy was shown in clinical trials to be safe and able to induce antitumor immune responses [1, 2]. However, durable clinical responses have only been observed in a minority of patients, underscoring the need to investigate new avenues of immunostimulatory DC vaccines for the generation of clinically relevant antitumor immune responses [3-6].

Moreover, clinical responses in DC trials are diverse and there is lack of immunologic readout systems that correspond with clinical outcome [7]. To date, DC vaccines are designed primarily to induce effective T cell responses, mostly ignoring the antitumor activity potential of natural killer (NK) cells [7, 8]. Indeed, NK cells have largely been neglected in the interpretation of the clinical outcome in DC vaccination, despite the fact that bidirectional crosstalk between NK cells and DC results in enhanced activation of both cell types and increases their antitumor activity [7]. In addition, NK cells can also directly act as helper cells for adaptive immunity in promoting effective T cell based antitumor responses [9]. Unfortunately, quantitative and qualitative abnormalities in the NK-cell compartment are frequently observed in different tumors, such as acute myeloid leukemia (AML) [10]. Therefore, combinatorial methods to awake or restore impaired NK cell functions may improve clinical outcome in DC vaccine trials [11].

Since its discovery two decades ago, interleukin (IL) 15 is subject of intense investigation for its immunostimulatory antitumor effects. In these years, IL 15 has become one of the most promising molecules for antitumor immunotherapy, due to its ability to stimulate both the innate and the adaptive arm of our immune system [12-14]. To exert its effects, IL 15 uses a unique transpresentation mechanism, whereby IL 15 bound to the α moiety of the IL 15 receptor (IL 15Rα) is being transpresented to the βγ chains of its receptor on neighboring cells [15-17]. In addition, IL 15 can bind to the IL 15 βγ receptor without forming a pre complex with IL 15Rα, although with a lower binding affinity [18]. Moreover, pre complexation of IL 15 with IL 15Rα results in an increase of the IL 15 half-life and therefore maximizes IL 15 activity *in vivo* [19, 20]. Therefore, combining IL 15 and IL 15Rα could possibly boost the *in vivo* antitumor functions of βγ expressing immune cells, such as NK cells and CD8^+^ T cells [21].

In this paper, we engineered human monocyte-derived mature DC to produce IL 15 and/or IL 15Rα using mRNA electroporation and studied their stimulatory effects on autologous NK cells. Combining these IL 15 ‘designer’ DC with NK cells results in enhanced activation of the latter, including the cytotoxic capacity against NK cell resistant tumor cells. We also show that IL 15 transpresentation is superior to IL-15 secretion for the NK cell stimulatory action. Subsequently, we validated the results in a human AML setting. Ultimately, this combinatorial approach and the subsequent (re)activation of NK cells may therefore be beneficial in the design of improved therapeutic DC-based vaccines for cancer patients.

## RESULTS

### Electroporation of DC with *IL-15* mRNA results in significant IL-15 secretion, but IL-15Rα is required for membrane expression of IL-15

**Figure 1:**
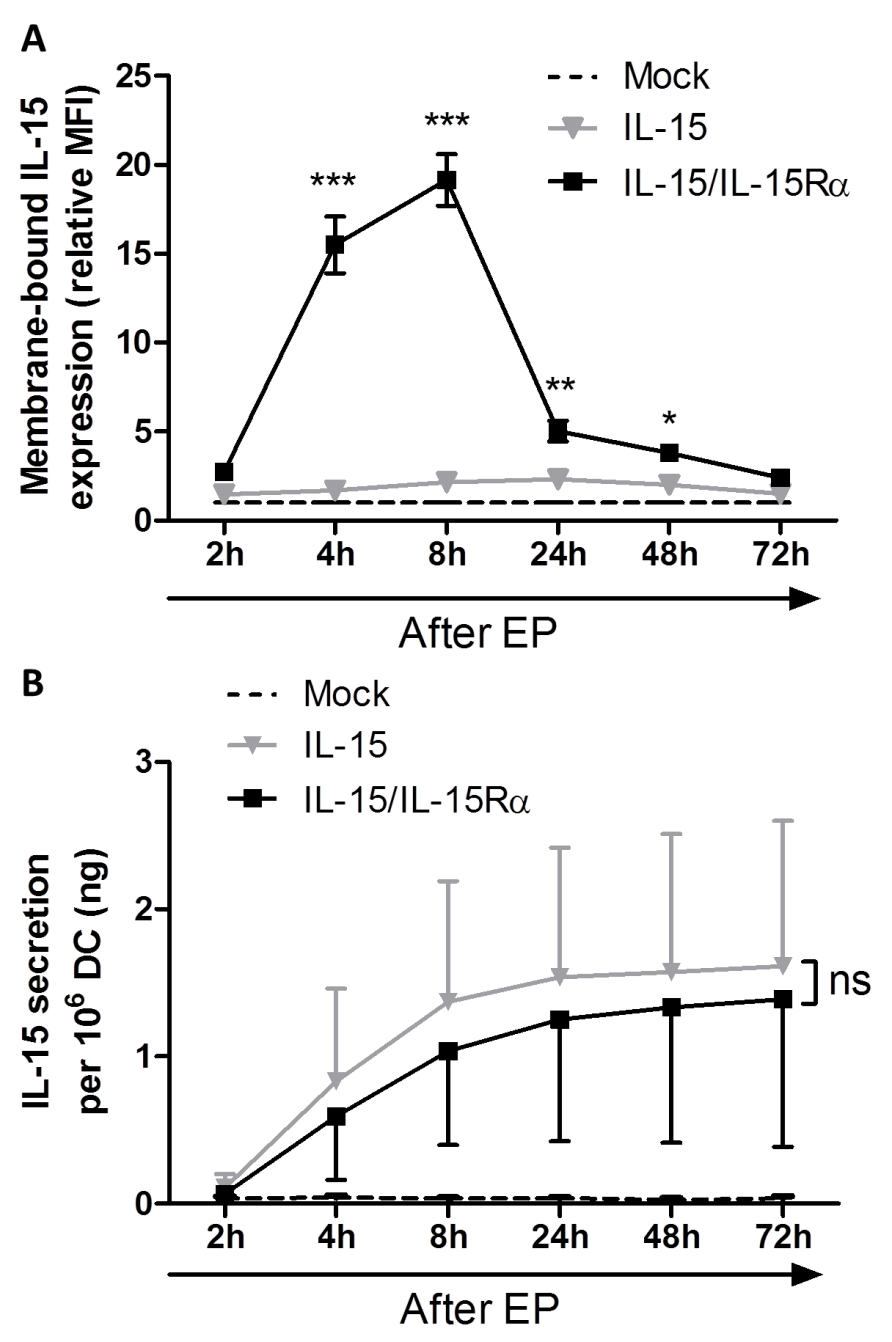
Interleukin-15 membrane expression and secretion of *IL-15* mRNA electroporated DC **A.** Membrane-bound IL-15 expression was determined by flow cytometric staining of mock EP DC (dashed black line), IL-15 EP DC (grey triangles) and IL-15/IL-15Rα EP DC (black squares) 2h, 4h, 8h, 24h, 48h and 72h after electroporation. Expression levels (MFI) were transformed to relative levels compared to those of the corresponding mock EP DC, which were set to one. Data are shown as mean (± SEM) for 3 independent donors. **B.** IL-15 secretion was quantified using an ELISA on the same EP conditions (mock EP DC, IL-15 EP DC and IL-15/IL-15Rα EP DC) and the same time points after electroporation (2h, 4h, 8h, 24h, 48h and 72h) as shown in figure 1A. Data are shown as mean (± SEM) for 6 independent donors. Statistical comparison was performed between IL-15 EP DC and IL-15/IL-15Rα EP DC at each time point. ns, not significant; *, *p* < 0.05; **, *p* < 0.01; ***, *p* < 0.001, two-way ANOVA with Bonferroni posthoc test. Abbreviations: EP; electroporation, MFI; mean fluorescence intensity, SEM; standard error of the mean.

As DC were modified to produce IL-15 and IL-15Rα in a transient manner, we sought to determine whether IL-15 was presented or secreted by the mRNA-electroporated DC and to analyze the expression kinetics of IL-15/IL-15Rα. Therefore we examined the supernatants and cells of transfected DC cultures (mock EP DC, IL-15 EP DC and IL-15/IL-15Rα EP DC) on different time points after mRNA electroporation. As compared with mock EP DC, no significant IL-15 membrane expression was observed on IL-15 EP DC (Figure 1A). However, electroporating IL-15Rα mRNA in addition to IL-15 mRNA resulted in a significant IL-15 expression on the membrane of IL-15/IL-15Rα EP DC as compared with IL-15 EP DC, with a peak expression at 8h after electroporation (*p* < 0.001). At 72h after electroporation, the IL-15 membrane expression almost completely disappeared (Figure 1A). Electroporating IL-15Rα mRNA only into DC (IL-15Rα EP DC) did not lead to any surface IL-15 expression (data not shown). Interestingly, regarding IL-15Rα expression, we demonstrate that this molecule is already present on monocyte-derived IL-4 DC and that the expression of IL-15Rα is only statistically significantly upregulated when both IL-15 and IL-15Rα mRNA are cotransfected into the DC (Supplemental Figure 1).

While IL-15 EP DC did not show significant membrane-bound IL-15, these DC secreted high levels of soluble IL-15, with the highest secretion between 2h and 8h after electroporation (Figure 1B). Despite the high donor variability, this production was even higher as compared with IL-15/IL-15Rα EP DC as seen in five out of six donors (Figure 1B). As seen for the IL-15 membrane expression, electroporating IL-15Rα mRNA only into DC did not display any IL-15 secretion (data not shown). For this reason, the IL-15Rα EP DC condition was not included in further experiments.

### IL-15 /IL-15Rα mRNA-electroporated DC induce phenotypic activation of NK cells

**Figure 2:**
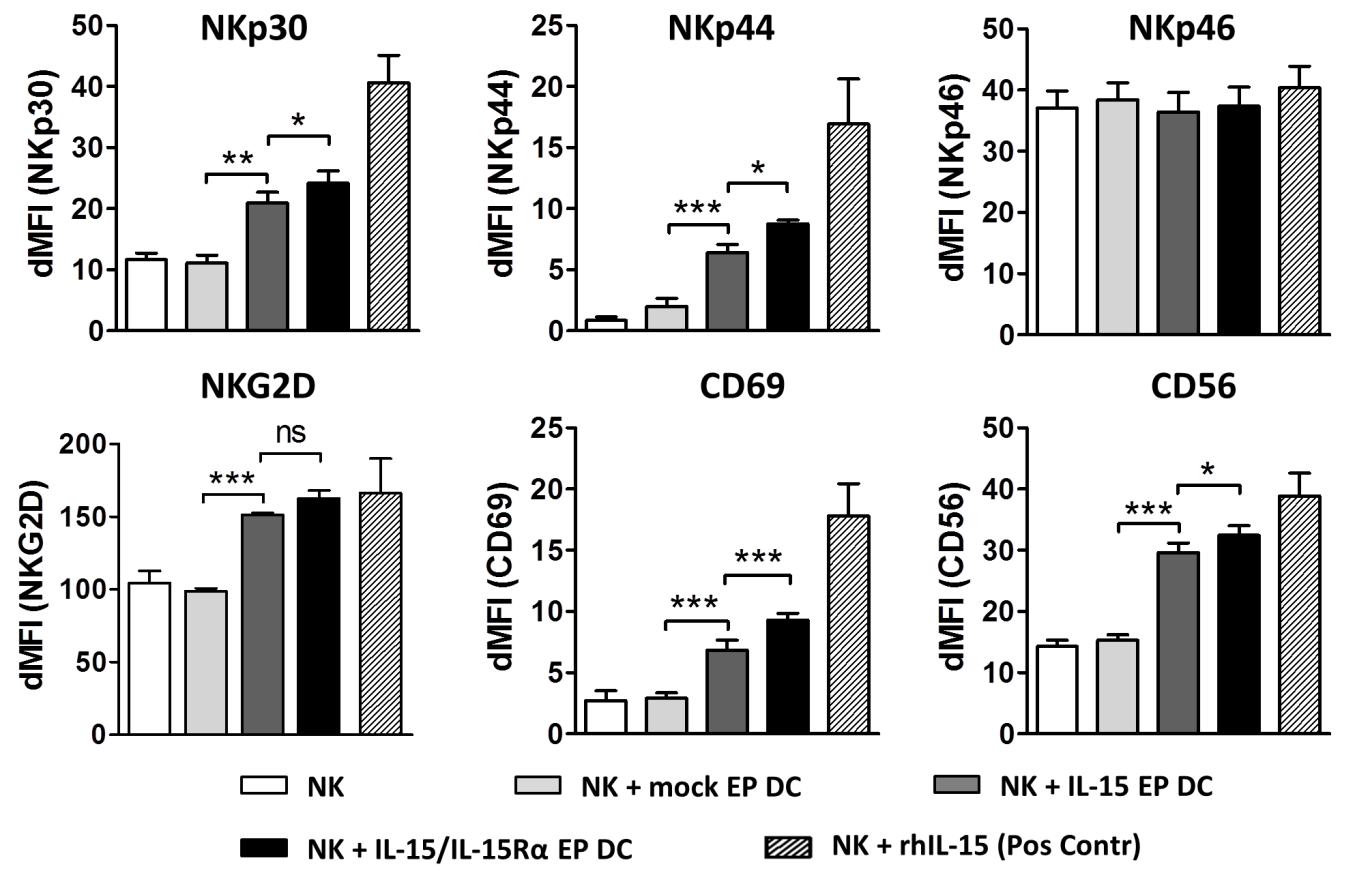
Phenotypic activation profile of DC- stimulated NK cells NK cells were cultured alone (white bars) or in coculture with mock EP DC (light grey bars), IL-15 EP DC (dark grey bars) or IL-15/IL-15Rα EP DC (black bars) in a NK/DC-ratio of 5:1. After 48h, NK-cell phenotype was determined using flow cytometry. Data, shown as dMFI (as compared to isotype controls), are depicted as mean (± SEM) for 3 independent donors. As a positive control, NK cells were stimulated with recombinant human IL-15 (1 ng/mL) for 48h before measuring phenotypic NK-cell activation (striped bars). ns, not significant; *, *p* < 0.05; **, *p* < 0.01; ***,*p* < 0.001, repeated measures one-way ANOVA with Bonferroni posthoc test. Abbreviations: EP; electroporation, dMFI; delta mean fluorescence intensity, SEM; standard error of the mean.

After a 48h coculture of IL-15 EP DC or IL-15/IL-15Rα EP DC with autologous NK cells, membrane expression of multiple typical NK-cell activation markers, including common natural cytotoxicity receptors, was observed. As shown in Figure 2, IL-15 produced by IL-15 EP DC (dark grey bars) led to a significant increase in the NK-cell membrane expression of NKp30 (*p* < 0.01), NKp44 (*p* < 0.001), CD69 (*p* < 0.001), NKG2D (*p* < 0.001) and CD56 (*p* < 0.001) as compared to NK cells alone (white bars) or in coculture with mock EP DC (light grey bars). Interestingly, when comparing the effect of IL-15 EP DC with IL-15/IL-15Rα EP DC (black bars) on the activation profile of NK cells, we detected an enhanced stimulation by IL-15/IL-15Rα EP DC as seen in the expression of NKp30 (*p* < 0.05), NKp44 (*p* < 0.05), CD56 (*p* < 0.05) and CD69 (*p* < 0.001).

### IL-15 EP DC and IL-15/IL-15Rα EP DC elevate production of lytic effector molecules and IFN-γ by NK cells

**Figure 3:**
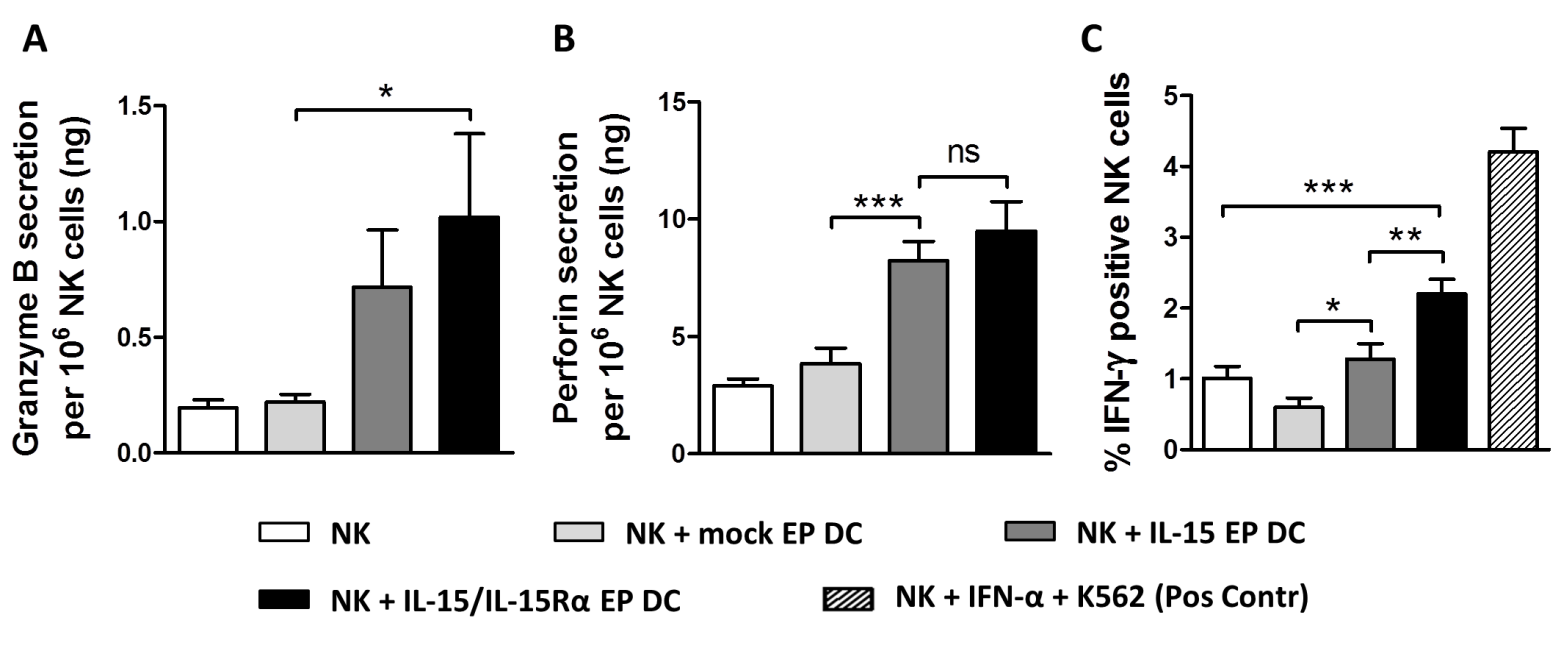
Granzyme B, perforin and IFN-γ production after NK/DC/tumor-cell coculture Granzyme B **A.** and perforin **B.** secretion are shown for 48h NK/Daudi (ratio 5:1, white bars) and NK/DC/Daudi (ratio 5:1:1, mock EP DC light grey bars, IL-15 EP DC dark grey bars, IL-15/IL-15Rα EP DC black bars) cocultures. Data are shown as mean (± SEM) for 5 (granzyme B) and 8 (perforin) independent donors, respectively. **C.** Intracellular IFN-γ, measured after 24h NK/DC/Daudi coculture (ratio 5:1:1), is shown for 6 independent donors. As a positive control, NK cells were stimulated with IFN-α (1000U/mL) for 48h and K562 cells for 4h (striped bar) before measuring intracellular IFN-γ. ns, not significant; *,*p* < 0.05; **,*p* < 0.01; ***, *p* < 0.001, repeated measures one-way ANOVA with Bonferroni posthoc test. Abbreviation: SEM; standard error of the mean.

From a mechanistic point of view to investigate the cytotoxic potential of DC-activated NK cells, supernatant of 48h NK/DC/Daudi cocultures was analyzed for the presence of granzyme B and perforin. Both proteins were undetectable in DC/Daudi cocultures (data not shown) but became apparent in the presence of NK cells (Figure 3A and 3B). While mock EP DC did not have an effect on the secretion of granzyme B and perforin by NK cells, their secretion was clearly elevated when IL-15 EP DC or IL-15/IL-15Rα EP DC were added to the NK cells and Daudi (Figure 3A and 3B). As seen for most NK-cell activation markers (vide supra) and killing of Daudi (vide infra), IL-15/IL-15Rα EP DC displayed a superior effect on NK cells as compared to IL-15 EP DC regarding granzyme B (Figure 3A) and perforin (Figure 3B) secretion, although not significantly different.

Furthermore, we observed intracellular IFN-γ production in NK/Daudi cocultures with or without mock EP DC, IL-15 EP DC or IL-15/IL-15Rα EP DC. While adding mock EP DC seemed to lower the production of IFN-γ by NK cells, IL-15 EP DC abrogated this reduction and even enhanced the IFN-γ production of NK cells (Figure 3C). Strikingly, adding IL-15/IL-15Rα EP DC to NK/Daudi cocultures resulted in almost a 2-fold increase of IFN-γ production by NK cells in comparison with IL-15 EP DC. This IFN-γ production was increased both in the CD56 dim and the CD56 bright NK-cell populations (data not shown). Notably, upon gating on the DC in 24h NK/DC/Daudi cocultures, none of the DC displayed any intracellular IFN-γ, regardless of the DC condition (mock EP DC, IL-15 EP DC or IL-15/IL-15Rα EP DC) (data not shown).

### IL-15/IL-15Rα EP DC increased the cytotoxic profile of NK cells by IL-15 signaling and partly in a contact-dependent manner

**Figure 4:**
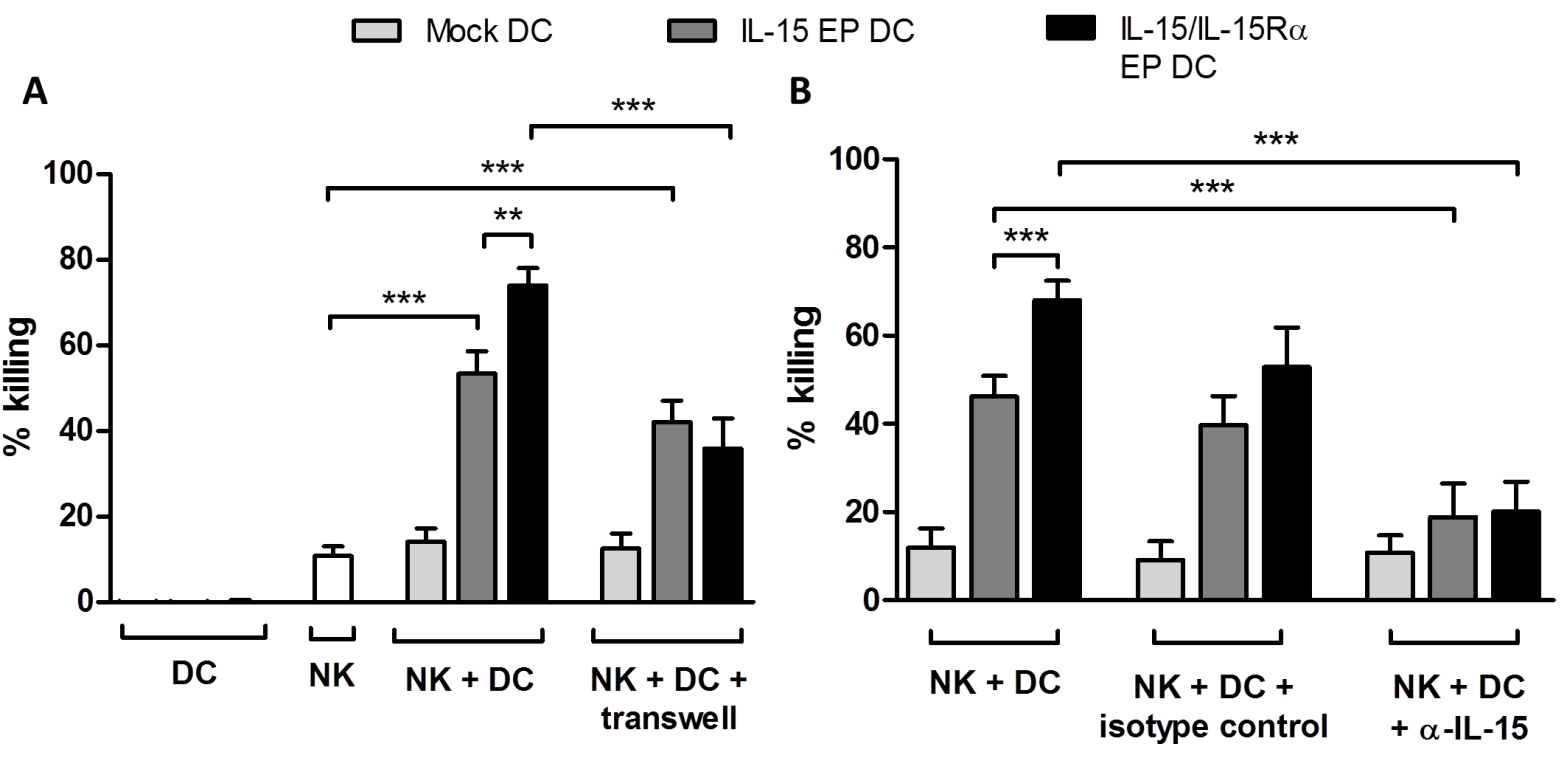
Cytotoxicity of NK/DC cocultures against Daudi cells The mean killing percentage (± SEM) of Daudi cells is shown for NK/Daudi (ratio 5:1, white bars), DC/Daudi (ratio 1:1, mock EP DC light grey bars, IL-15 EP DC dark grey bars, IL-15/IL-15Rα EP DC black bars) and NK/DC/Daudi (ratio 5:1:1, mock EP DC light grey bars, IL-15 EP DC dark grey bars, IL-15/IL-15Rα EP DC black bars) cocultures based on a 4h flow cytometric cytotoxicity assay following 44h NK-cell and/or DC cocultures. **A.** In some conditions, a transwell insert (pore size of 4 μM) was added before starting NK/DC cocultures. These data are shown as mean (± SEM) for 5-9 independent donors. **B.** 1h prior to the addition of NK cells, anti-IL-15 neutralizing IgG or corresponding IgG isotype control antibody (100 μg/4x105 DC) was added to DC. These data are shown as mean (± SEM) for 4-8 independent donors. **, *p* < 0.01; ***, *p* < 0.001, one-way ANOVA with Bonferroni posthoc test. Abbreviation: SEM; standard error of the mean.

Cocultures of IL-15 EP DC and autologous NK cells resulted in an elevated killing of Daudi as compared to the basal level of NK cell-mediated tumor-cell killing (Figure 4A; *p* < 0.001). This tumoricidal capacity was even further enhanced if IL-15/IL-15Rα EP DC and NK cells were put in coculture (*p* < 0.01). As a positive control, NK cells alone or in combination with DC were cocultured with leukemic cells (K562) which are known to be killed easily by NK cells (Supplemental Figure 2). In order to check whether this effect was contact-dependent, transwell experiments were performed to separate DC and NK cells. The superior tumor-cell killing effect of IL-15/IL-15Rα EP DC/NK-cell cocultures leveled out to the cytotoxic level of IL-15 EP DC/NK-cell cocultures using transwells, indicating that a contact-dependent IL-15 transpresentation mechanism is involved in the superior killing effect (Figure 4A, *p* < 0.001).

Furthermore, to determine if the increased killing capacity of NK cells stimulated with IL-15 EP DC or IL-15/IL-15Rα EP DC was correlated with the IL-15 expression and/or secretion, IL-15 was blocked by adding an anti-IL-15 neutralizing antibody before coculture. As shown in Figure 4B, neutralizing IL-15 resulted in a statistically significant reduction of tumor-cell killing (*p* < 0.001), pointing to an important role for IL-15 in the observed killing effect.

### Confirmation of increased cytotoxic profile of DC-activated NK cells using immune cells of cancer patients *ex vivo*

**Figure 5:**
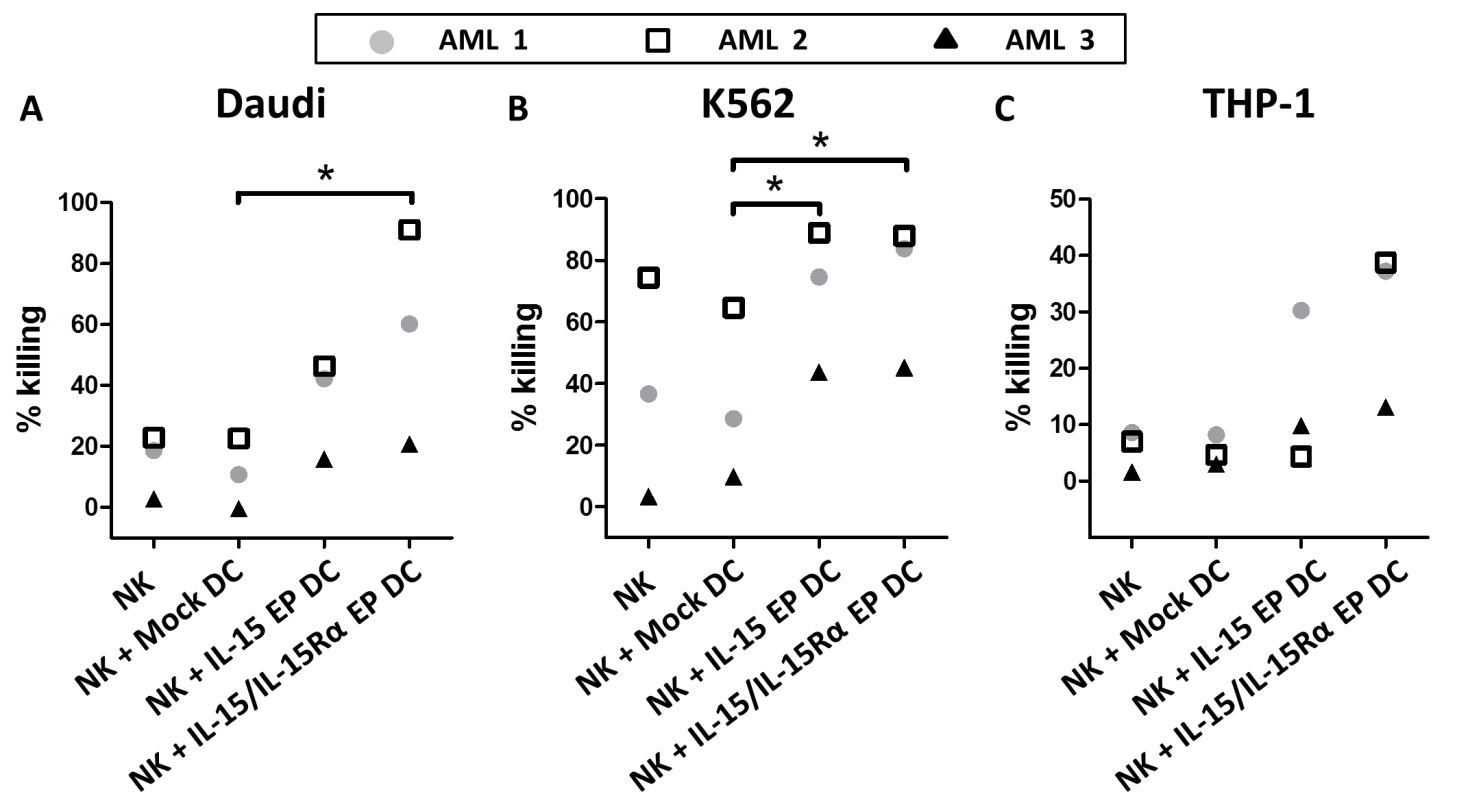
Validation of the cytotoxic profile of DC-activated NK cells against tumor cells for AML patients in remission The killing percentage of **A.** Daudi, **B.** K562 and **C.** THP-1 is shown for NK/DC/tumor-cell (ratio 5:1:1) cocultures of three different AML patients in remission based on a 4h flow cytometric cytotoxicity assay following 44h NK/DC cocultures. AML 1 and AML 2 are already a few years in remission, while patient AML 3 was tested shortly after consolidation. *, *p* < 0.05, one-way ANOVA with Bonferroni posthoc test.

To confirm the killing capacity of DC-activated NK cells against tumor cells, monocyte-derived DC and autologous NK cells from three AML patients in remission were used. As observed for all three AML patients, coculturing IL-15 EP DC and NK cells increased the killing against the NK-cell resistant Daudi cell line dramatically (Figure 5A). Replacing IL-15 EP DC by IL-15/IL-15Rα EP DC even boosted this effect. The enhanced killing for AML-patients 1 and 2 (which were already in remission for some years) was comparable to that of healthy donors, while for AML patient 3 who only recently was brought into remission after consolidation therapy overall less tumor killing was observed. For patient 3 however, IL-15 EP DC and IL-15Rα EP DC induced tumoricidal activity of NK cells that was absent if no DC were added. For all 3 patients, similar effects were seen for killing of the leukemic cell lines K562 and THP-1 compared to killing of Daudi cells (Figure 5B and 5C). Interestingly, the increased killing of NK cells in coculture with IL-15 EP DC or IL-15/IL-15Rα EP DC could also be shown using DC and NK cells derived from a glioblastoma multiforme patient pointing towards a more general immunostimulatory approach that could be applied to both hematological and solid tumors (Supplemental Figure 3).

## DISCUSSION

Due to the ability of IL-15 to stimulate both the innate and the adaptive arm of the immune system, including in the context of antitumor immunity, IL-15 was labeled as the immunotherapeutic drug with the greatest potential for broad usage in cancer therapy [22]. However, IL-15 can cause substantial systemic toxicity, particularly when administered on a daily basis [23, 24]. In addition, pharmaceutical companies are not enthusiastic to produce GMP-grade cytokines such as IL-15 following some early clinical disappointments with systemic cytokine-based immunotherapy [25]. To avoid the systemic delivery of IL-15, but profit fully of its immunostimulatory properties, IL-15 can also be transfected in immune-competent cells for subsequent production and secretion, as shown in this study. From a GMP perspective it is easier to obtain clinical grade IL-15 mRNA than protein, so mRNA-based transfection (e.g. through electroporation) [26] of IL-15 mRNA in such cells circumvents the obstacle of the scarcely available clinical grade protein IL-15. Noteworthy, the half-life of IL-15 is less than one hour, limiting its bioactivity *in vivo*, but the half-life and stability of IL-15 can be enhanced by binding to IL-15Rα, which occurs in the so-called IL-15 transpresentation process [19, 20]. In this report, we tried to tackle both problems by simultaneously electroporating IL-15 and IL-15Rα mRNA into DC. With this innovative designer DC-based strategy, we aimed to achieve more immune-stimulatory DC for future use in DC vaccination protocols.

Co-electroporating IL-15 and IL-15Rα mRNA into DC, results on one hand in a high membrane expression and on the other hand in secretion of IL-15. More specifically, IL-15 and the α-moiety of the IL-15 receptor can form a pre-complex internally and these complexes are subsequently transported to the cell membrane, where IL-15Rα can present IL-15 to neighboring cells. If one of both components is not available, no stable pre-complex can be formed and hampers IL-15 transpresentation on the cell membrane [20, 27-29]. We hypothesized that IL-15Rα mRNA electroporation of IL-15-producing cells, such as monocytes and DC, can result in elevated IL-15 expression on the membrane [30]. Unfortunately, no increase in membrane-bound IL-15 was detected as compared with mock EP DC, questioning endogenous IL-15 production in monocyte-derived DC differentiated in the presence of GM-CSF and IL-4 or trafficking of endogenous IL-15 via the secretory pathway in these cells. However, changing the DC maturation cocktail from TNF-α/PGE_2_ into a combination of IFN-γ and a Toll-like receptor agonist, can result in a high production of IL-15 by these DC [31], which may offer an alternative to the use of IL-15 mRNA.

Our designer DC can present IL-15 to neighboring IL-15Rβγ-expressing cells, such as NK cells. Previously it was shown that DC-derived IL-15 is able to stimulate proliferation and activation of these immune effector cells in both a virus- and tumor-related setting [32-38]. Our results show that IL-15-producing DC are able to (1) increase phenotypic NK-cell activation, (2) elevate lytic effector molecules and IFN-γ secretion of DC- stimulated NK cells and (3) increase killing of tumor cells after NK/DC cocultures. In addition, when IL-15 is pre-complexed with IL-15Rα, superior stimulatory effects on NK cells were observed likely due to a higher binding affinity with the βγ-moiety of the IL-15 receptor [16, 20, 27, 28, 39, 40]. Our observations corroborate these findings, including enhanced killing of tumor cells if IL-15 is presented by IL-15Rα, as seen by us using healthy donor and cancer patient material. AML patients in remisson and glioblastoma multiforme patients are very interesting target groups for DC vaccination to eliminate residual cancer cells and to prevent or to postpone relapse [41-44]. Interestingly, for the AML patient that had just undergone consolidation therapy, the designer DC could even restore NK cell killing activity that was dysfunctional in the absence of DC. Due to technical reasons, we used frozen NK cells in all the NK-cell assays in this report, which might affect their killing capacity [45]. This NK cell-freezing obstacle is not present when applying DC vaccination in the *in vivo* setting, suggesting that even better immunostimulatory results of our designer DC might be expected.

As reported by Guo and colleagues, IFN-γ-production is essential to IL-15/IL-15Rα-induced immunocytotoxicity, as IFN-γ knock-out mice did not show any improvement in the activation of NK cells after treatment with IL-15/IL-15Rα complexes [46]. On top, based on our IFN-γ data, we show that IL-15/IL-15Rα-expressing dendritic cells are able to activate all subsets of NK cells, both CD56 dim and CD56 bright. Interestingly, transwell inserts between NK cells and DC abrogate the superior effect of IL-15/IL-15Rα complexes, favoring the engagement of IL-15 transpresentation in a contact-dependent manner [39].

Although IL-15/IL-15Rα EP DC are promising candidates for DC-based vaccination in cancer immunotherapy, the tumor suppressive environment can impede its powerful antitumor effects. Therefore, combining our immunostimulatory DC with other antitumor therapies, such as immune checkpoint inhibitors, could further improve the efficacy of both our DC vaccine and other current immunotherapies. Furthermore, to promote their clinical application, our DC can be loaded with different kinds of tumor antigens and since the expansion and survival effects of IL-15 on CTLs are antigen-independent [47], our DC vaccine can be used against a broad range of tumor types.

Thus, in this report we show that our novel IL-15/IL-15Rα designer DC are promising candidates to improve DC vaccination strategies in the battle against cancer, based on two different key points. First of all, IL-15Rα chaperones IL-15 to be expressed on the membrane of DC, potently stimulating DC-mediated NK-cell activation [39]. This IL-15/IL-15Rα transpresentation mechanism lies at the root why DC-derived IL-15 was found to be superior to rIL-15 in eliciting durable therapeutic antitumor immunity [37]. Secondly, our IL-15/IL-15Rα mRNA electroporation technique ensures a transient effect of IL-15/IL-15Rα complexes, which is in favor of the activation of NK cells as compared to prolonged stimulation by IL-15/IL-15Rα complexes. Remarkably, prolonged stimulation by IL-15/IL-15Rα complexes leads to a marked accumulation of NK cells with considerably impaired activation, cytotoxicity, and proliferative activity, and an altered balance of activating and inhibitory receptors [40]. In conclusion, we show that IL-15Rα plays a pivotal role in IL-15-based NK-cell activation and that the use of IL-15/IL-15Rα mRNA-engineered DC is an attractive approach to improve antitumoral NK-cell activity in DC-based vaccination strategies.

## MATERIALS AND METHODS

### Ethics statement and cell material

This study was approved by the Ethics Committee of the University of Antwerp (Antwerp, Belgium) under the reference number 13/49/500. Experiments were performed using blood samples from anonymous donors provided by the Antwerp branch of the Red Cross Blood Transfusion Center (Mechelen, Belgium) or from patients with AML or glioblastoma multiforme obtained from the hematological/oncological division of the Antwerp University Hospital (Edegem, Belgium). AML patients were only selected when they were in remission and excluded when they previously received a hematopoietic stem cell transplantation. The human NK-cell resistant Daudi cell line (gift from Dr. K. Thielemans of the Free University of Brussels (VUB)) and the leukemic cell lines K562 (ATCC; Ref: CCL-243) and THP-1 (ATCC; Ref: TIB-202) were maintained in Iscove's Modified Dulbecco's Medium (IMDM; Invitrogen; Ref: 21980-032) supplemented with 10% fetal bovine serum (FBS; Invitrogen; Ref: 10270-106). The adherent glioblastoma cell lines U87-MG (CLS; Ref: 300367) and LN-229 (ATCC; Ref: CRL-2611) were maintained in Dulbecco's Modified Eagle Medium (DMEM; Invitrogen; Ref: 10938-025) supplemented with 10% FBS. All cell lines were maintained in logarithmic growth phase at 37°C in a humidified atmosphere supplemented with 5% CO_2_.

### mRNA

The human *OSP-IL-15* gene [48] was generated into a pST1 vector by gene-ART (Life Technologies), putting it under the control of a T7 promoter and providing it with a poly(A)tail [49]. The human *IL-15Rα* gene was a kind gift of Dr. B. Weiner (University of Pennsylvania, Philadelphia, USA) and was subcloned into a pST1 vector. mRNA transcripts were generated using a mMessage mMachine T7 *in vitro* transcription kit (Life Technologies; Ref: AM1344) according to the manufacturer's protocol.

### IL-15 designer DC generation

DC were generated as described previously [50] with minor adaptations specific for the IL-15 designer DC. Briefly, positively selected CD14^+^ monocytes were differentiated into mature DC according to a 7-day culture protocol. [50] After 7 days, DC were harvested and electroporated without mRNA (mock EP DC), with 10 μg *OSP-IL-15* mRNA (IL-15 EP DC), 10 μg *IL-15Rα* mRNA (IL-15Rα EP DC) or a combination of 10 μg *OSP-IL-15* mRNA and 10 μg *IL-15Rα* mRNA (IL-15/IL-15Rα EP DC) in 200 μL Opti-MEM reduced serum medium without phenol red (Life Technologies, Ref: 11058-021).

### NK cells

Untouched resting CD3^−^CD56^+^ NK cells were isolated from the thawed CD14^−^ peripheral blood lymphocyte fraction, which was frozen at day -7 in freezing medium consisting of Roswell Park Memorial Institute 1640 (RPMI; Invitrogen, Ref: 21875-034) supplemented with 20% FBS and 10% dimethyl sulfoxide (Sigma-Aldrich, Ref: D2650), after overnight resting at 37°C. NK cells were isolated using the human negative selection NK-cell isolation kit (Miltenyi Biotec, Ref: 130-092-657) according to the manufacturer's instructions. NK-cell purity and viability was at least 95% as determined on a FACScan flow cytometer (BD) following staining with fluorescein isothiocyanate (FITC)-conjugated anti-CD3 (BD; Ref: 21810033 and phycoerythrin (PE)-conjugated anti-CD56 (BD; Ref: 345812) monoclonal antibodies (mABs), and propidium iodide (PI; Invitrogen, Ref: P3566), respectively.

### Membrane immunophenotyping

DC were membrane-stained with monoclonal anti-IL-15-PE antibody (R&D, Ref: LIT0713051) 2h, 4h, 8h, 24h, 48h and 72h after electroporation of the DC and analyzed on a FACScan flow cytometer (BD). In other experiments, purified NK cells were cocultured at a 5:1 ratio with autologous DC in IMDM + 10% FBS directly after DC electroporation and membrane-stained with anti-CD11c-V450 (BD; Ref: 560369), anti-NKp30-AF360 (BD; Ref: 558408), anti-NKp44-PE (BD; Ref: 558563), anti-NKp46-APC (BD; Ref: 557940), anti-NKG2D-PE (BD; Ref: 557940), anti-CD56-FITC (BD; Ref:345811) and anti-CD69-APC-Cy7 (BD; Ref: 557756) mABs 48h after initiation of cocultures. 7-aminoactinomycin D (7-AAD; BD; Ref: 51-68981E) was used to distinguish between viable and dead cells. Samples were measured on a FACSAria II flow cytometer (BD).

### Cytokine secretion assays

Secretion of IL-15 by DC was determined in supernatant 2h, 4h, 8h, 24h, 48h and 72h after electroporation using a human IL-15 enzyme-linked immunosorbent assay (ELISA; eBioscience; Ref: 88-7158-88) according to the manufacturer's protocol. The secretion of granzyme B and perforin was determined in supernatant of 48h NK/DC/tumor-cell cocultures (cocultured directly after DC electroporation) using a human granzyme B (Cell Sciences; Ref: CKH329) and perforin (Diaclone; Ref: 851.860.005) ELISA kit, respectively, according to the manufacturer's protocol. Both ELISA assays were measured on a Victor 3 multilabel counter (Perkin Elmer).

### Intracellular staining

IFN-γ production was measured using a flow cytometric-based intracellular staining assay after 24h NK/DC/tumor-cell cocultures. Briefly, tumor cells were added to 6h NK/DC cocultures (cocultured directly after DC electroporation), followed 1h later by addition of a protein transport inhibitor (GolgiPlug; 5μL/mL) (BD; Ref: 555029). After overnight incubation at 37°C and 5% CO_2_, cells were membrane-stained with fluorochrome-labeled anti-CD3 (BD; Ref: 560835), anti-CD11c (BD; Ref: 560369), anti-CD56 (BD; Ref: 345812) and LIVE/DEAD Aqua (Invitrogen; Ref: L34957). Next, cells were fixed and permeabilized using the Foxp3/transcription factor staining buffer kit (eBioscience; Ref: 00-5523-00) according to the manufacturer's instructions with minor adaptations. Briefly, cells were fixed by adding fix/perm working solution during 1h at 4°C, followed by two washing steps with permeabilization buffer. Permeabilized cells were stained with anti-IFN-γ antibody (BD; Ref: 554700) for 1h at 4°C. Samples were measured on a FACSAria II flow cytometer (BD).

### Cytotoxicity assay

The killing capacity of DC and NK cells against tumor cells was determined using a flow cytometry-based protocol as described previously with minor modifications [4, 42, 50, 51]. Briefly, tumor cells were labeled prior to coculture with PKH67 Green Fluorescent Cell Linker dye (Sigma-Aldrich; Ref: MIDI67-1KT) according to the manufacturer's protocol. PKH67^+^ tumor cells were cultured alone or added to 48h-cocultures of NK cells and DC, which were cocultured directly after DC electroporation, for 4h at an NK/DC/tumor-cell ratio of 5:1:1. After 4h coculture, samples were stained with annexin V-APC (BD; Ref: 550474) and PI (BD; Ref: P3566) followed by acquisition on a FACSAria II flow cytometer. Cytotoxicity was calculated based on the viability (annexin V^−^/PI^−^) of PKH67^+^ tumor cells using the following equation: % killing = 100% - (% annexin V^−^/PI^−^ tumor cells with effector cells/% annexin V^−^/PI^−^ tumor cells without effector cells). In specific experiments, anti-IL-15 neutralizing IgG mAB (R&D; Ref: MAB647) or corresponding IgG isotype control mAB (R&D; Ref: MAB002) was added to cultures of DC 1h prior to the addition of NK cells for 48h. To determine the cell-contact dependence of NK cells and DC regarding IL-15 signaling, transwell inserts with a pore size of 0.4 μM (BD; Ref: 353095) were used at the day of coculturing the cells. The transwell inserts were removed before adding tumor cells 48h after coculture.

### Statistical analysis

Flow cytometry data were analyzed using FlowJo version 10.0.6 (Treestar). GraphPad Prism 5 software was used for graphing and statistical calculations. Statistical analysis was performed using the (repeated-measures) one-way or two-way analysis of variance with Bonferroni post-hoc test, where appropriate. Results were considered statistically significant when *p* < 0.05.

## SUPPLEMENTARY MATERIAL FIGURES


