# Supplementary material for: Transpresentation of interleukin-15 by IL-15/IL-15Rα mRNA-engineered human dendritic cells boosts antitumoral natural killer cell activity
Source: Oncotarget. 2015 Dec 9;6(42):44123–33. doi: 10.18632/oncotarget.6536 (PMC4792546; doi:10.18632/oncotarget.6536)
Supplement: Supplementary file 1 [file oncotarget-06-44123-s001.pdf]

Transpresentation of interleukin-15 by IL-15/IL-15Rα mRNA-engineered human dendritic cells boosts antitumoral natural killer cell activity

Supplementary Material

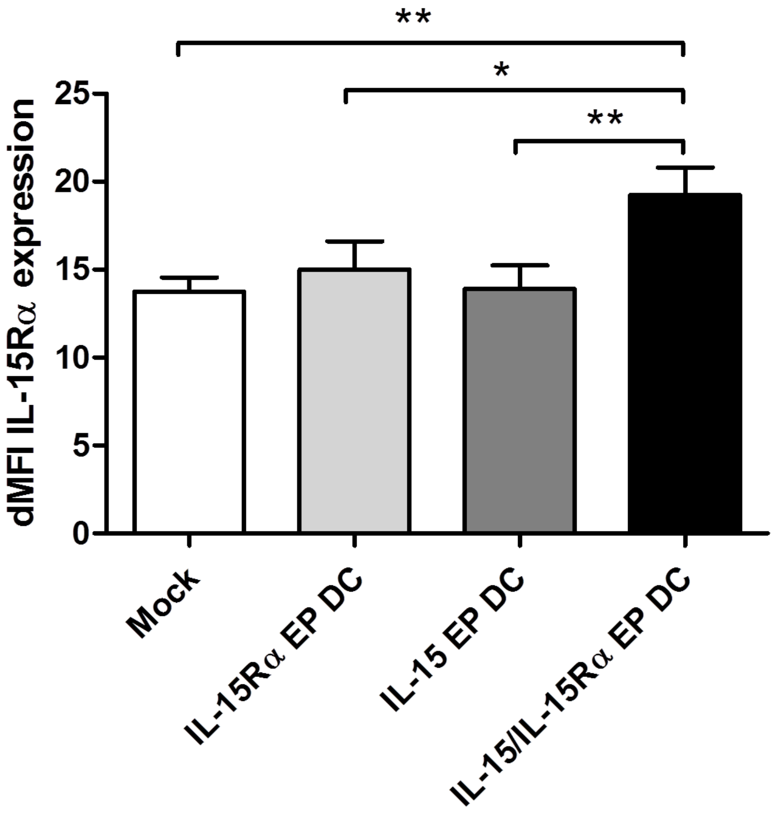

Supplemental Figure 1: Interleukin-15 receptor alpha membrane expression of *IL-15* mRNA electroporated DC

Membrane-bound IL-15Rα expression (dMFI as compared with isotype controls) was determined by flow cytometric staining of mock EP DC (white bar), IL-15Rα EP DC (light grey bar), IL-15 EP DC (dark grey bar) and IL-15/IL-15Rα EP DC (black bar) 4h after electroporation. *, $p < 0.05$; **, $p < 0.01$, repeated measures one-way ANOVA with Bonferroni posthoc test. Abbreviations: EP; electroporation, dMFI; delta mean fluorescence intensity.

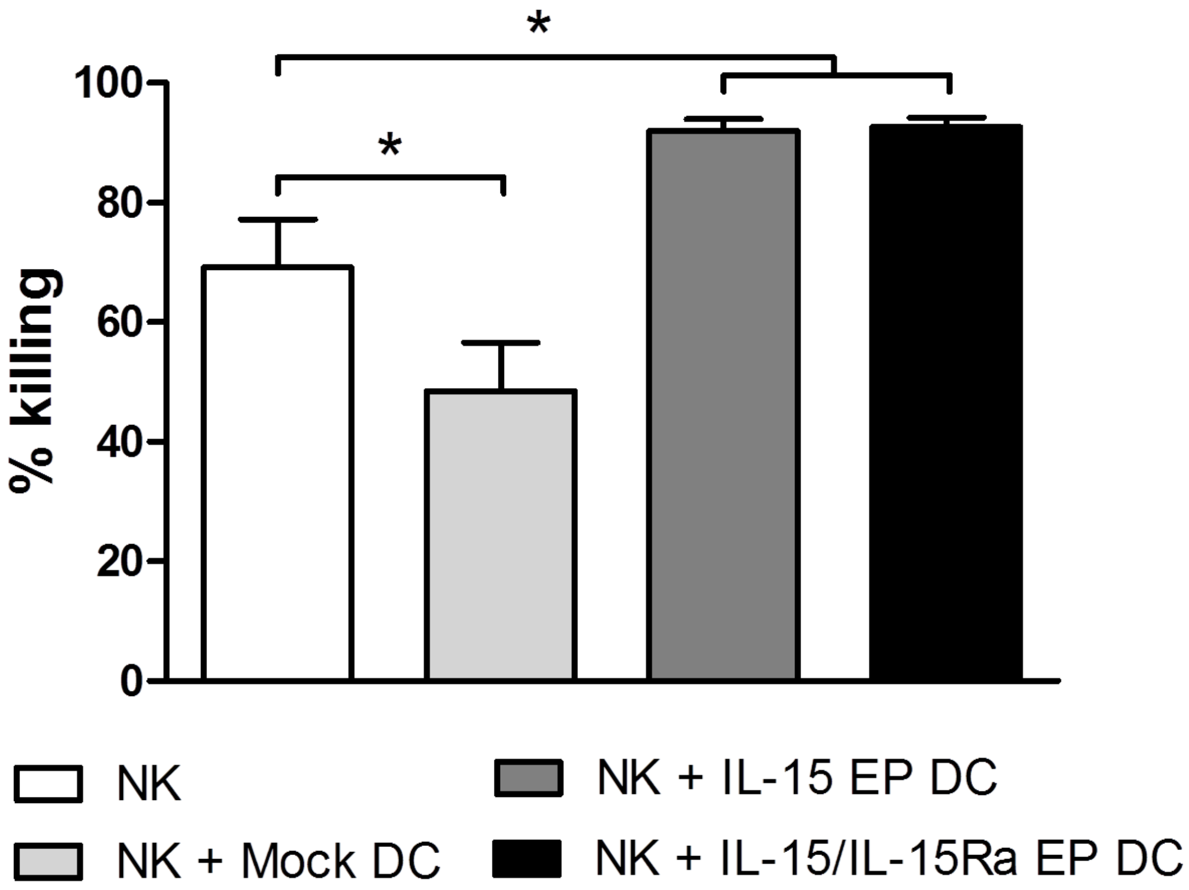

Supplemental Figure 2: Cytotoxicity of NK/DC cocultures against K562 cells

The mean killing percentage (± SEM) of K562 cells is shown for NK/K562 (ratio 5:1, white bars) and NK/DC/K562 (ratio 5:1:1, mock EP DC light grey bars, IL-15 EP DC dark grey bars, IL-15/IL-15Rα EP DC black bars) cocultures based on a 4h flow cytometric cytotoxicity assay following 44h NK-cell and/or DC cocultures. Data are shown as mean (± SEM) for 7 independent donors. *, $p < 0.05$, one-way ANOVA with Bonferroni posthoc test. Abbreviation: SEM; standard error of the mean.

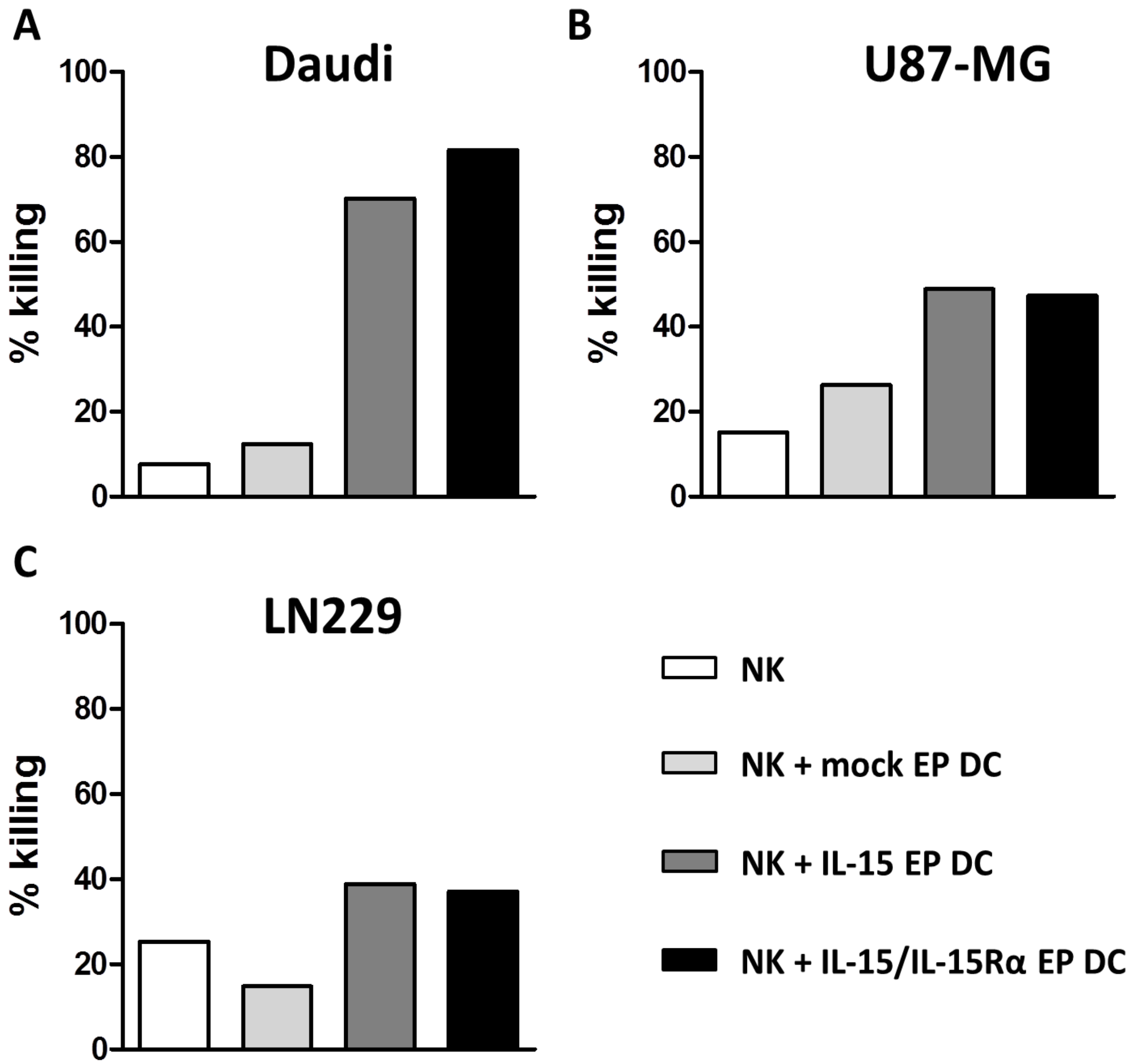

Supplemental Figure 3: Validation of the cytotoxic profile of DC-activated NK cells against tumor cells using immune cells from a glioblastoma multiforme patient

The killing percentage of **A** Daudi, **B** U87-MG and **C** LN229 is shown for NK/tumor-cell (ratio 5:1, white bars) and NK/DC/tumor-cell (ratio 5:1:1, mock EP DC light grey bars, IL-15 EP DC dark grey bars, IL-15/IL-15Rα EP DC black bars) cocultures based on a 4h flow cytometric cytotoxicity assay following 44h NK-cell and/or DC cocultures.
